# Exposure of the SH-SY5Y Human Neuroblastoma Cells to 50-Hz Magnetic Field: Comparison Between Two-Dimensional (2D) and Three-Dimensional (3D) In Vitro Cultures

**DOI:** 10.1007/s12035-020-02192-x

**Published:** 2020-11-24

**Authors:** Claudia Consales, Alessio Butera, Caterina Merla, Emanuela Pasquali, Vanni Lopresto, Rosanna Pinto, Maria Pierdomenico, Mariateresa Mancuso, Carmela Marino, Barbara Benassi

**Affiliations:** 1 Division of Health Protection Technologies, ENEA-Casaccia Italian National Agency for New Technologies, Energy and Sustainable Economic Development, Via Anguillarese 301, 00123 Rome, Italy; 2 Experimental Medicine and Surgery, University of Rome Tor Vergata, 00133 Rome, Italy

**Keywords:** Extremely low-frequency magnetic field, SH-SY5Y, *In vitro*, 3D culture, MicroRNAs

## Abstract

We here characterize the response to the extremely low-frequency (ELF) magnetic field (MF, 50 Hz, 1 mT) of SH-SY5Y human neuroblastoma cells, cultured in a three-dimensional (3D) Alvetex^®^ scaffold compared to conventional two-dimensional (2D) monolayers. We proved that the growing phenotype of proliferating SH-SY5Y cells is not affected by the culturing conditions, as morphology, cell cycle distribution, proliferation/differentiation gene expression of 3D-cultures overlap what reported in 2D plates. In response to 72-h exposure to 50-Hz MF, we demonstrated that no proliferation change and apoptosis activation occur in both 2D and 3D cultures. Consistently, no modulation of *Ki67*, *MYCN*, *CCDN1*, and *Nestin, *of invasiveness and neo-angiogenesis-controlling genes (*HIF-1α*, *VEGF*, and *PDGF*) and of microRNA epigenetic signature (miR-21-5p, miR-222-3p and miR-133b) is driven by ELF exposure. Conversely, intracellular glutathione content and *SOD1* expression are exclusively impaired in 3D-culture cells in response to the MF, whereas no change of such redox modulators is observed in SH-SY5Y cells if grown on 2D monolayers. Moreover, ELF-MF synergizes with the differentiating agents to stimulate neuroblastoma differentiation into a dopaminergic (DA) phenotype in the 3D-scaffold culture only, as growth arrest and induction of *p21*, *TH*, *DAT*, and *GAP43* are reported in ELF-exposed SH-SY5Y cells exclusively if grown on 3D scaffolds. As overall, our findings prove that 3D culture is a more reliable experimental model for studying SH-SY5Y response to ELF-MF if compared to 2D conventional monolayer, and put the bases for promoting 3D systems in future studies addressing the interaction between electromagnetic fields and biological systems.

## Introduction

The culturing of primary neurons and neuronal-like cancer cells on two-dimensional (2D) surfaces represents the conventional *in vitro* experimental model for both oncologic and neurodegenerative disorders. The limitations of such growing conditions are recently emerging, as 2D cultures lack the complex anatomical and functional connectivity of the neuronal network that underlies both the physiological and pathological condition [1–3]. To provide a more functional, structural, and biochemical system that might closely resemble the *in vivo* environment, different three-dimensional (3D) matrices have been developed, including microporous polystyrene scaffolds, fibrin matrices, agarose, matrigel, and collagen hydrogels [4–7]. Moreover, although still in its infancy, the bioengineering of 3D brain organoids (based on of stem cell–derived, self-organizing 3D cell cultures) is recently emerging to introduce additional degrees of complexity [8, 9].

Comparison of cell growth in standard 2D monolayer cultures and 3D matrix highlighted clear phenotypic differences in terms of cellular surface area, stress fiber distribution, cellular migration and adhesions, neurite growth, and dimensions, as well as in protein/gene expression and epigenetic markers [10–12]. More importantly, cellular response to drugs and ionizing radiations has been shown to be significantly affected by culture conditions [13–15]. Glioblastoma stem cells are more radio-resistant if cultured in 3D conditions (similar to what was observed *in vivo*) than corresponding 2D cultures, consistent with the data reported in other non-neuronal histotypes [16, 17].

In this context, the exposure to the electromagnetic fields (EMFs) of cells grown in 3D cell systems is gaining a growing interest (mainly for tissue regeneration purposes), although the experimental data are still very limited [18–20]. A few reports characterized the response to the extremely low-frequency (ELF) magnetic field (MF) in both primary bovine chondrocytes and mesenchymal stem cells grown in 3D cultures to stimulate chondrogenesis and cartilage maturation [21, 22], or in epidermal stem cells seeded in collagen sponge scaffolds for improving wound healing [23].

However, there are no experimental data assessing the response of neuronal and neuroblastoma cells—grown in 3D matrices—to the ELF-MFs. This issue appears particularly relevant, as the mechanism(s) underlying the interaction between the neuronal cell and the ELF-MF are still a matter of debate. Epidemiological data suggest a possible association between occupational and environmental exposure to ELF-MF with the increased incidence of neurodegenerative diseases, mainly amyotrophic lateral sclerosis (ALS) and Alzheimer’s disease (AD) [24, 25], and childhood brain tumors [26, 27]. On the other side, ELF-MF might promisingly be applied for therapeutic purposes in brain disorders [28, 29]. In this context, we recently reported that 50-Hz MF affects the iron homeostasis in the *in vitro* SOD1^G93A^ ALS model, thus providing preliminary evidence for the exploitation of an EMF-based therapy in ALS pathology [30].

All the *in vitro* findings addressing the issue of neuronal and neuroblastoma response to ELF-MF—including those from our group [30–34]—have been carried out in conventional 2D cultures. Therefore, the possibility to develop an *in vitro* study in a 3D matrix is challenging, to put the bases for further investigations in a model system that might be as close as possible to the *in vivo* condition.

We thus aimed at characterizing the effect triggered by the exposure to ELF-MF (50 Hz, 1 mT) in the SH-SY5Y human neuroblastoma cells grown (under both proliferative and differentiating conditions) [35, 36] in a commercial, polystyrene-based 3D scaffold (Alvetex^®^) [12, 17, 37, 38], compared to the conventional 2D monolayer culture. By applying the exposure conditions previously optimized and characterized by our group [30–34], we focused on different biological endpoints, ranging from the proliferation and differentiation pathways to the microRNA (miR)-related epigenetic changes; we also verified some key redox homeostasis modulators, as ELF-MF exposure has been extensively documented to exert pro-oxidant properties in various *in vitro* and *in vivo* models, including neuronal cells where the exposure to ELF-MF stimulates ROS generation and impairs the antioxidant defense [31–34, 39–42].

## Materials and Methods

### Chemicals

Culture media, serum and supplements, trypsin-EDTA, and phosphate-buffered saline (PBS) were obtained from Euroclone (Milan, Italy). 4′,6-Diamidine-2′-phenylindole dihydrochloride (DAPI), ethylenediamine tetraacetic acid (EDTA), formalin, Neutral Red, paraffin, phorbol 12-myristate 13-acetate (PMA), poly-D-lysine, propidium iodide (PI), all-trans retinoic acid (RA), RNAse A, Triton X-100, and trypan blue solution (0.4%) were purchased from Sigma-Aldrich (Milan, Italy). Ethanol was obtained from CARLO ERBA Reagents (Milan, Italy).

### Cell Culture Conditions in 2D Monolayers and 3D Scaffold

Human SH-SY5Y neuroblastoma cells were purchased from the European Collection of Cell Culture, cultured in complete Dulbecco’s modified Eagle’s medium/Ham’s F12 (DMEM/F12 (50:50 mix, Euroclone), supplemented with 10% heat-inactivated fetal bovine serum, 2 mM L-glutamine, 100 μg/ml streptomycin, and 100 units/ml penicillin, and kept in culture up to 15 passages. The cells were maintained at 37 °C in a 5% CO_2_ atmosphere in air and routinely trypsinized and plated at 4 × 10^4^/cm^2^ on flasks. Cell counting was performed at the hemocytometer following trypan blue staining exclusion.

The conventional 2D monolayer culture was carried out by plating cells in 12-well plates. For differentiation experiments, plates were pre-coated with poly-L-lysine (10 μg/ml) for 2 h and washed twice in PBS before seeding cells.

For 3D cultures, the commercial 200-μm-thick polystyrene scaffolds (Alvetex^®^, ReproCELL, Durham, UK) were used (Online Resource 1a) and manipulated according to the manufacturer’s instructions (https://www.reprocell.com). To render the scaffold hydrophilic, inserts were first submerged in 70% ethanol for 10 min, then washed twice with sterile water, and incubated with poly-L-lysine (10 μg/ml) for 2 h. After coating, the inserts were washed with PBS, placed in a 12-well plate, and finally incubated in serum-containing media for 2 h at 37 °C and 5% CO_2_. Before starting the study, different setup tests in the 3D scaffolds have been carried out to optimize the cellular density, the visualization of cell by staining with Neutral Red (Online Resource 1b), and the yield of cell amount, proteins, and RNA from scaffolds for further analyses.

Cell retrieval from Alvetex^®^ scaffold has been carried out according to the manufacturer’s indications. In brief, inserts have been unclipped, and the scaffold discs carefully removed using flat-ended forceps; each disc has been gently washed in PBS and cut into quarters with a sterile scalpel to increase the exposed surface area. Disc quarters have been moved to a sterile 15-ml centrifuge tube containing 5 ml of trypsin-EDTA and incubated at 37 °C on a shaking platform set to 100 rpm for 10 min to help cell detaching from the support.

In each experiment, 3 × 10^5^ cells were seeded in either a 12-well plate (3.5 ml of complete medium) (2D cultures) or in Alvetex^®^ scaffold placed in a 12-well plate (covered by 3.5 ml of complete medium) (3D cultures). Both 2D and 3D cultures have been always plated simultaneously, to allow direct comparison of each biological endpoint.

### Exposure System

The ELF-MF exposure system consists of two couples of square coils (two coils for each sub-system, arranged coaxially in Helmholtz configuration), as previously detailed [31]. Briefly, the coils are connected to a Variac (40NC) for voltage feeding and current circulation within the cable turns. The two systems are used for exposure to the induction magnetic field (B-field) at power frequency (50 Hz) and for sham exposure of the biological samples at the same time, thus allowing blind experimental conditions. The coil double wire configuration is used for sham exposure implementation, which allows to obtain a null B-field by using currents flowing in opposite directions. The B-field produced by these systems, at the operating frequency of 50 Hz, was set at a root mean square (RMS) amplitude of 1 mT for a supplied current of 3.4 A. The sham exposures were performed at a residual B-field amplitude of about 0.3 μT (RMS), representing the background field emitted by the incubator electronics. B-field measurements were performed at the center of the exposure volume of each couple of coils (20 × 20 × 10 cm^3^) with an isotropic B-field probe (ELT400, Narda, Pfullingen, Germany), in both sham and real exposure configurations. To assess the B-field homogeneity within the exposure setup and the induced electric field (E-field) values within the culture samples contained in Petri dishes, numerical simulations were carried out using a finite element method [31].

Globally, our experimental and numerical data guaranteed a high homogeneity (95%) for B-field in the exposure volume, and the proper positioning of the biological samples for rigorously controlled and repeatable exposure conditions [31]. In order to guarantee the temperature stability (37 °C) inside the incubators, a refrigerating system, consisting of water from two separate thermostatic baths and circulating in plastic tubes surrounding the coils, was set up to prevent heating due to ohmic losses, thus maintaining the temperature of both B-field and sham-exposed samples at the temperature of 37.0 ± 0.2 °C. The temperature was monitored in the exposure volume of each incubator by two T-thermocouple probes (SENSORTEK Inc., Clifton, NJ, USA), placed in a dummy Petri dish and in air.

### Exposure System Dosimetry

**Fig. 1:**
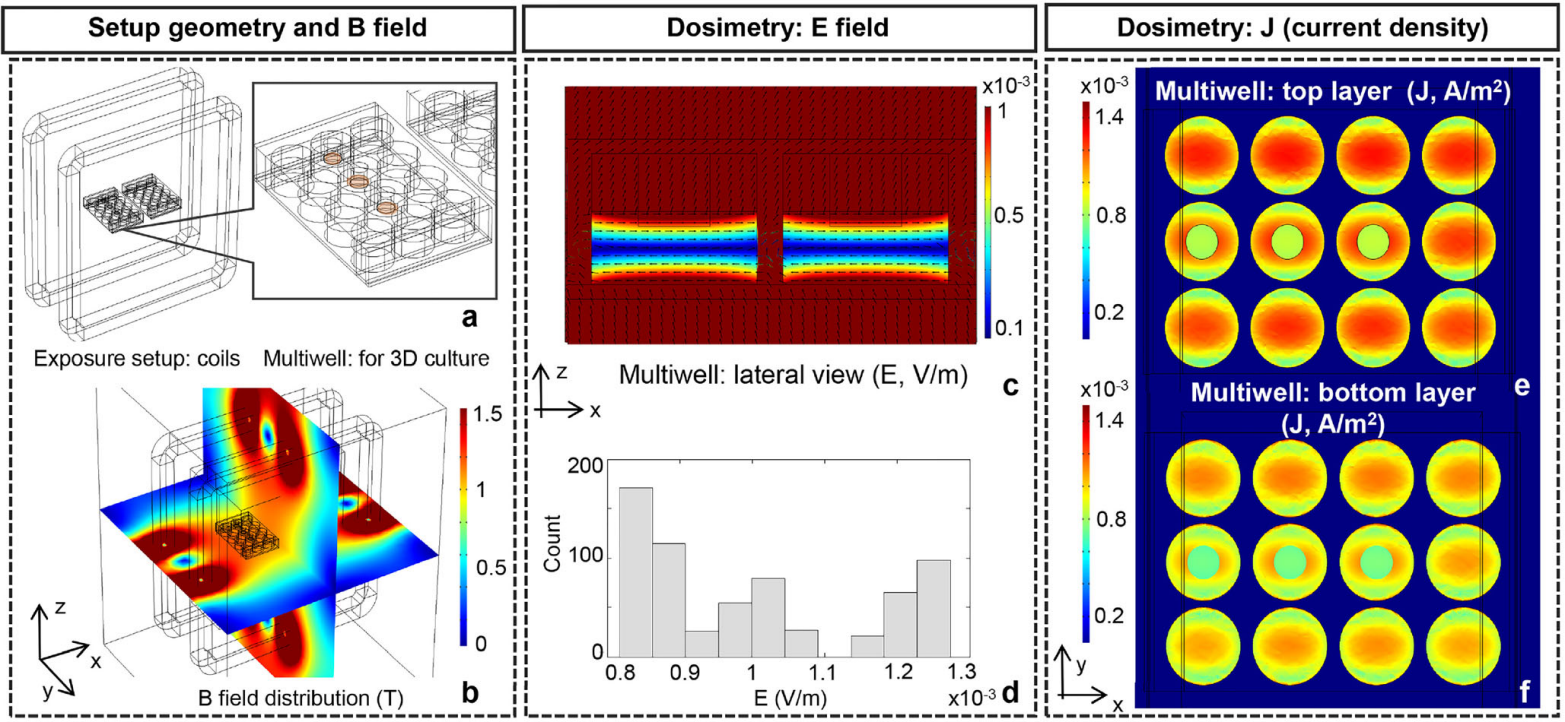
Exposure system and dosimetry. **a** Configuration of the exposure coil simulations including a couple of 12 multi-well; the 3D scaffolds are shown in color. **b** Spatial distribution of the simulated B-field (50 Hz) is presented, the level of 1 mT is reached in the multi-well volume. **c** Spatial distribution of the induced E-field (50 Hz) is shown on a front view. **d** Bar distribution of the averaged induced E-field on all the exposed multi-well. The spatial distributions of the induced current densities (50 Hz) are shown at the top (**e**) and bottom (**f**) layer of the multi-well

The dosimetric description of the experimental setup was carried out to define levels of induced current densities (J) and E-field within each exposed sample and to assess their homogeneity in the 3D culture conditions. Hence, the exposure coils were modeled using their real dimensions as already reported in [31, 34] and shown in Fig. 1a. In order to ensure the highest homogeneity of the induced E-field and J at the level of the polystyrene scaffolds, the lines of the magnetic flux density need to be parallel to the biological sample holder. Thus, the two coils were placed side by side and equidistant to a couple of the 12 multi-well containers. Each well was filled with 3.5 ml of biological solution (cells plus medium). The multi-well plates were simulated as dielectric Plexiglas containers, with a relative permittivity of 2.8, while the biological solution has a relative permittivity of 78 and a conductivity of 1.5 S/m both assessed as suggested in [31, 34]. The polystyrene scaffolds, considered in simulations as a separate layer of 0.95 mm of thickness, have dielectric characteristics equal to 60 and 1 S/m for permittivity and conductivity respectively (https://www.reprocell.com).

The simulated geometry is fully sketched in Fig. 1a, with the inset showing the multi-well and the polystyrene scaffolds in color. The simulations were carried out using the numerical solver COMSOL Multiphysics (v. 5) in the frequency domain. The same current of 3.4 A, effectively used to feed the experimental system, was chosen for the simulations and applied to the stimulating coils. Electric boundary conditions terminated the simulation domain to simulate the presence of the cell incubator. Numerical dosimetry of the macroscopic biological environment permitted defining homogeneity of the J and E-field distributions in the whole biological sample and in the polystyrene scaffolds to conduct controlled and high-quality biological experiments.

### Cell Culture Exposure to MF and Treatments

The exposure experiments were carried out by exposing cells to B-field at 50-Hz MF, 1-mT (RMS) intensity, representing the low-action level for occupational exposure [43]. Cell exposure to either sham or MF has been always performed under blind conditions.

Twenty-four hours after plating, proliferating SH-SY5Y cells underwent a continuous (72 h) exposure to either sham or ELF-MF, as previously detailed by our group [30–34]. For dopaminergic (DA) differentiation experiments, cells were treated with 10 μM retinoic acid (RA, 3 days), followed by 50 nM phorbol 12-myristate 13-acetate (PMA, for further 3 days) in reduced serum condition (5%) [44, 45]. The differentiation plus MF exposure schedule has been previously optimized [31, 33] and summarized in Fig. 6a; neuroblastoma cells were first treated with RA (for 3 days, without any exposure) then administered with PMA (for further 3 days) in combination to either ELF-MF or sham exposure.

### Proliferation Assay by WST-1 Assay

The assessment of cell proliferation was carried by the colorimetric Cell Proliferation Reagent WST-1 kit (Roche, Roche Diagnostics GmbH, Mannheim, Germany).

For 3D culture, the test has been performed, adapting the specific Alvetex^®^ indications to the WST-1 protocol (https://www.reprocell.com). In brief, scaffolds have been washed in PBS prior to running the assay, to remove the medium. The discs have been gently removed from the scaffold with flat-ended forceps, dip in PBS, placed into a new 12-well plate, and treated with 1 ml of WST-1 reagent (diluted 1:10 in fresh medium) at 37 °C and 5% CO_2_ for 3 h. In conventional 2D cultures, cells have been washed with PBS, treated with 1 ml of WST-1 reagent (diluted 1:10 in fresh medium), and processed as 3D cultures. After incubation, the medium was collected and analyzed with a spectrophotometer at an absorbance of 440 nm.

### Evaluation of cell Cycle Distribution by DNA Content Analysis

The analysis of DNA content for evaluation of the cell cycle distribution was carried out as previously described [31]. Adherent cells were harvested by trypsinization and collected with floating ones; the pool was washed twice in PBS, then fixed in ice-cold ethanol 80% (1 × 10^6^ cells/ml) overnight. An aliquot of the suspension (at least 5 × 10^5^ cells) was then washed twice in PBS and stained with PI (50 μg/ml) in a mix containing RNAse A (50 μg/ml), Triton X-100 (0.1%), and EDTA (0,1 mM) in PBS, in the dark, for 60 min at room temperature, then immediately analyzed. A FACScan flow cytometer (Becton Dickinson, Bedford, MA, USA), equipped with a 488-nm argon laser, was used for the flow cytometric analyses. The evaluation of cell cycle distribution by DNA content analysis was performed by the FlowJo software^®^.

### Hematoxylin and Eosin Staining, Immunohistochemistry, and Immunofluorescence

Both H&E and immunohistochemistry (IHC)/immunofluorescence (IF) immunostaining have been carried out in paraformaldehyde-fixed 2D cultures and in formalin-embedded 3D slices, the latter according to Alvetex^®^ protocols (https://www.reprocell.com).

To evaluate cell morphology, 2D cultured cells were plated and grown on cover glasses, fixed for 30 min at room temperature in 4% paraformaldehyde (Electron Microscopy Sciences, Hatfield, PA, USA) in PBS, then washed and stained with hematoxylin and eosin. 3D scaffolds were instead formalin-fixed and paraffin-embedded according to standard methods, and then 3-μm-thick paraffin sections were stained with H&E [34].

The assessment of proliferative and apoptotic index was carried out by IHC of 3D cultures sections and IF of 2D cultures, as previously described [46]. Antibodies used were cleaved caspase-3 (≠9661; polyclonal, Cell Signaling Technology, Inc., Danvers, MA; 1:100), PCNA (monoclonal 1:100; Millipore, Billerica, MA, USA), and Ki67 (polyclonal, Novocastra Laboratories, Newcastle, UK; 1: 800). DAPI was used at 0.5 μg/ml final concentration.

### RNA Extraction, Reverse Transcription, and Gene Expression Analysis

Total RNA was extracted from samples by Trizol^®^ (Invitrogen, Thermo Fisher Scientific, Waltham, MA, USA) followed by spin-column elution, also including a DNAse digestion step (Direct-zol^TM^ RNA miniPrep, Zymo Research, Irvine, CA, USA). In 2D cultures, cells were harvested by trypsinization, and Trizol^®^ was added to the pellet. For 3D cultures, the Alvetex^®^ manufacturer’s protocol was followed (https://www.reprocell.com). In brief, each scaffold was washed by gentle immersion in PBS using flat-ended forceps and transfer to a clean 12-well plate. After the addition of Trizol^®^ (600 μl), the scaffold was placed on a rotating platform (100 rpm) for 10 min at room temperature. The lysate was further homogenized by passaging up and down through a 20-gauge needle 10 times, using a sterile plastic syringe, and then processed according to Direct-zol^TM^ RNA miniPrep protocol.

The amount and purity of the extracted RNA were evaluated by a fiber-optic spectrophotometer (Nanodrop ND-1000, NanoDrop Technologies, Wilmington, DE, USA) calculating the 230/260 and the 260/280 absorbance ratios. Five hundred nanograms of total RNA was reverse-transcribed to cDNA with random primers by TaqMan^®^ Reverse Transcription Reagent (Applied Biosystems, Thermo Fisher Scientific), according to the manufacturer’s indications. The analysis of the gene expression was carried out with 1 μl of cDNA using the SYBR Green master mix (Applied Biosystems) and an Eco™ Real-Time PCR System (Illumina, San Diego, CA, USA). All reactions were run in triplicate and the relative abundance of the transcripts was calculated by normalizing to the ribosomal protein 18s (18s) expression, applying the 2^-ΔΔCt^ method [47]. PCR primers were designed by NCBI-Primer Blast free software (https://www.ncbi.nlm.nih.gov/tools/primer-blast), according to gene sequences available in the UCSC database (https://genome.ucsc.edu), and selected to amplify an exon-intron-exon region (≤ 200 bp) to exclude genomic contamination. PCR primers were synthesized by Eurofins Scientific (Luxembourg). The complete list of primer sequences is reported in Online Resource 2.

### microRNA Expression Analysis

The analysis of mature miRNA expression was carried out on total RNA, as previously described [33]. Ten nanograms of total RNA was retro-transcribed by the miRcury LNA universal RT microRNA kit (Exiqon, Denmark); cDNA was diluted 1:80 and amplified by the miRcury LNA Sybr green master mix and miR-specific LNA PCR primer sets (Exiqon), according to the manufacturer’s instructions. All reactions were run in quadruplicate and the relative abundance of each specific microRNA was normalized to RNU1A1 small nucleolar RNAs, by applying the 2^−ΔΔCt^ method [47].

### Reduced Glutathione Content Assay

The intracellular content of reduced glutathione (GSH) has been measured by a colorimetric assay (Bioxytech GSH-400; Oxis International, Inc., Los Angeles, CA, USA), as previously described [48]. Briefly, cells have been harvested by trypsinization from either 2D or 3D cultures (the latter, according to Alvetex^®^ cell retrieval protocol as previously detailed), lysated in 500 μl of ice-cold metaphosphoric acid working solution (0.5 g/l in water) and centrifuged at 3000*g*, 4 °C for 10 min. The upper clear aqueous layer has been collected, kept at 0–4 °C for the assay according to the manufacturer’s protocol (to be performed within 1 h from lysis), and absorbance was read at 400 nm. Glutathione concentration has been calculated according to GSH standard curve, previously obtained by reading different standard GSH samples at 400 nm, 25 °C.

### Statistical Analysis

The variations of samples values are reported as mean ± S.D. calculated in *N* = 3 independent experiments. The statistical differences were analyzed trough the KailedaGraph statistical software (Synergy Software, Reading, PA, USA) by applying either (i) the non-parametric Mann-Whitney *U* test for unpaired groups (when two groups have been compared) or (ii) the factorial ANOVA test followed by the post hoc Newman-Keuls/Tukey (in the case of multiple comparisons). *P* values < 0.05, indicated in the figures with the asterisk symbol, were considered statistically significant.

## Results

### Two-Dimensional Dishes Versus Three-Dimensional Alvetex^®^ Scaffolds: Culture Condition Does Not Affect the Growing Phenotype of Proliferating SH-SY5Y Human Neuroblastoma Cells

**Fig. 2:**
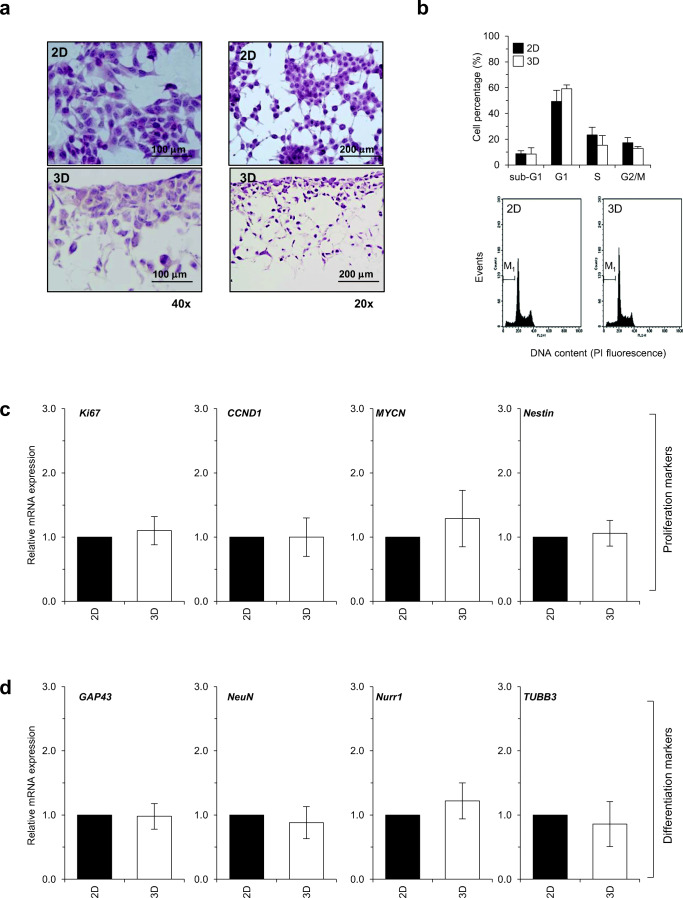
The growing phenotype of proliferating SH-SY5Y human neuroblastoma cells (at day 4 of culture) is not affected by the culturing conditions. **a** Cell morphology evaluated by hematoxylin/eosin staining of paraformaldehyde-fixed (2D) and paraffin-embedded (3D) samples. Magnification and scale bars are reported in each panel. **b** Cell cycle distribution (FACS analysis of DNA content by PI staining; M_1_ mark highlights the sub-G_1_ population). **c, d** Gene expression analysis (normalized to 18s expression by real-time PCR), carried out in SH-SY5Y cells grown in both 2D cultures and 3D scaffold. H&E staining and FACS histograms reported in the figures are each from a typical experiment representative of three independent ones. Values are means ± SD (*N* = 3 independent experiments)

We first characterized the growing features of SH-SY5Y human neuroblastoma cells, cultured on either 2D monolayer or 3D Alvetex^®^ scaffold, in order to assess whether the culture conditions might affect their phenotype. Cell morphology and confluency were evaluated during the exponential phase (4 days in culture), in cells plated at a density of 3 × 10^5^cells/dish (2D) and 3 × 10^5^cells/scaffold (3D). Neuroblastoma cells exhibited a ~ 50–60% confluency in conventional monolayer cultures, whereas they are well-distributed in depth in the polystyrene scaffold, displaying a lower confluency deep in the 3D matrix (Fig. 2a). However, no change in cell shape was detectable between the two culture conditions. As shown by flow cytometric analysis of PI-stained cells (Fig. 2b), cell cycle distribution was not affected by culture conditions and no change in the sub-G_1_ percentage was detected between 2D and 3D cultures. This was consistent with the WST-1 proliferation index assessment that showed no change between 2D and 3D cultures (data not shown here, see Fig. 6c).

To better characterize the growing phenotype, gene expression profile of a set of proliferation and neuronal differentiation markers was carried. The expression level of pro-proliferative *Ki67* and cyclin D1 (*CCND1*) genes [49, 50], as well as of *MYCN* and *Nestin* (controlling neuroblastoma aggressiveness [51, 52]), did not undergo any significant change in cells, independent of either 2D or 3D condition (Fig. 2c). In terms of mature neuronal markers, we tested the growth-associated protein (*GAP43*), neuronal nuclei protein (*NeuN*), Nur-related factor 1 (*Nurr1*), and beta-3 tubulin (*TUBB3*) [35, 36], and we did not detect any statistically significant variation between 2D monolayer and 3D scaffold (Fig. 2d).

### 50-Hz MF Does Not Affect Proliferation, Apoptosis, and Angiogenetic Biomarkers in Proliferating SH-SY5Y Cells Grown in Both 2D and 3D Cultures

We next exposed proliferating SH-SY5Y cells (grown in both 2D and 3D culture conditions) to 50-Hz MF (1 mT) for 72 h (continuous exposure), according to conditions previously characterized by our group [30–34].

As first, we quantified the current density (J) and the E-field induced by the ELF-MF exposure in the 3D cultures, by numerical simulations [34]. In order to verify that the induced quantities are correct, the level of the magnetic (B) field generated by the coils was monitored, and its spatial distribution was reported in Fig. 1b. The multi-well is exposed in a region of homogeneous B-field at an intensity of 1 mT, as done during the real biological experiments. Therefore, E-field spatial distribution at 50 Hz induced by a 1-mT B-field on the *xz* plane (at the center of the multi-well) is shown in Fig. 1c. Observing a *xz* plane, it is clear that the polystyrene scaffolds are exposed in a very homogeneous fashion (Fig. 1c). This is confirmed by looking at the quantitative bar diagram of the E-field distribution reported in Fig. 1d. The induced E-field in the Alvetex® polystyrene scaffold has an average value of around 1 mV/m with an inhomogeneity of the distribution equal to 15.5%. This means that all cells are exposed in a very similar fashion during the exposure. Inhomogeneity of E-field is quantitatively evaluated as the % ratio between standard deviation and mean value, evaluated over all the polystyrene scaffold volume. Similar trends are reported for the induced J, spatial distributions at the bottom and at the top planes (*yx*) of the polystyrene scaffold, as shown in Fig.1e–f. The inhomogeneity of J resulted in around 15%, still confirming the high homogeneity of the exposed region, the average value of J resulted being around 1 A/m^2^ in scaffolds (Fig.1e–f).

**Fig. 3:**
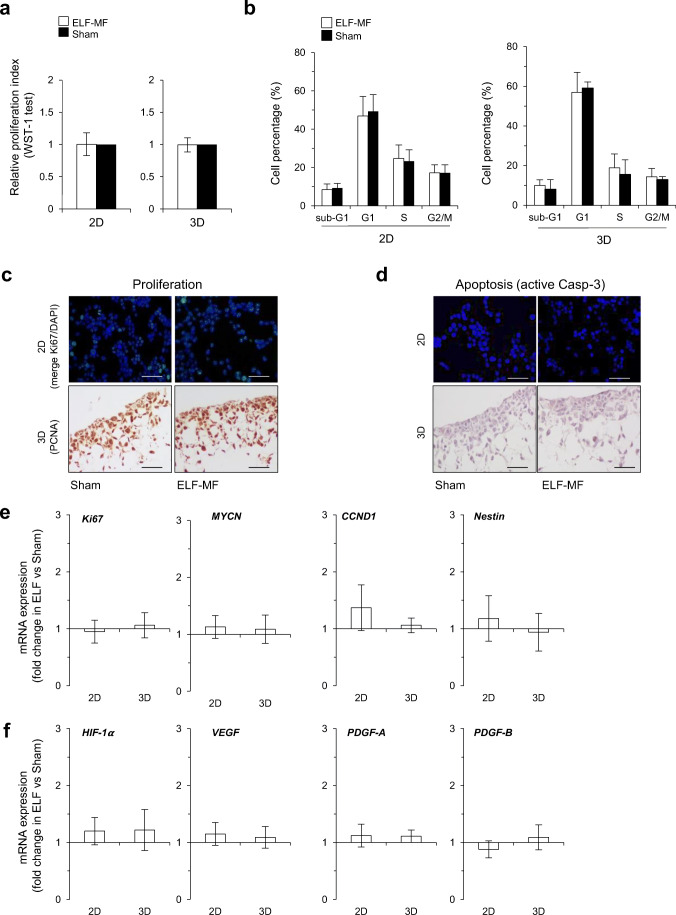
50-Hz MF does not affect proliferation, apoptosis, and angiogenetic biomarkers in proliferating SH-SY5Y cells grown in both 2D and 3D cultures. Cells were exposed for 72 h to 50-Hz (1 mT) MF and characterized according to the following endpoints: **a** relative proliferation index (by WST-1 test) and **b** cell cycle distribution (FACS analysis of DNA content by PI staining). Values represent means ± SD (*N* = 3 independent experiments). **c** Evaluation of proliferative ability carried out by IF in 2D conventional cultures (merge Ki67/DAPI staining) and by IHC in 3D Alvetex^®^ scaffolds (PCNA staining). Scale bar: 200 μm. **d** Assessment of apoptosis percentage by active-caspase-3 antibody staining. Scale bar: 200 μm. **e**, **f** Gene expression analysis of proliferation, invasiveness, and neo-angiogenesis-related transcripts (normalized to 18s expression by real-time PCR), performed in 2D- versus 3D-cultured SH-SY5Y cells. The values reported represent the mean fold change (± SD) in mRNA expression of ELF- versus sham-exposed cells (chosen as a reference value). No significant difference has been observed (*N* = 3 independent experiments)

At the experimental level, we reported no change in the proliferation index (WST-1 test, Fig. 3a) and in the distribution of cell cycle phases (Fig. 3b, and Online Resources 3a) in ELF versus sham-exposed neuroblastoma cells, in both conventional monolayer and 3D matrix. Accordingly, the immunostaining of Ki-67 expression (in 2D cultures) and proliferating cell nuclear antigen (PCNA) (in 3D slices) confirmed the absence of any statistically significant variation of proliferation markers in ELF compared to sham-exposed cells (Fig. 3c, and Online Resources 3b). These findings were corroborated by gene expression profiling of *Ki-67*, *CCND1*, *MYCN*, and *Nestin* genes that did not undergo any modulation in response to ELF-MF under both 2D and 3D culture conditions (Fig. 3e). Also, in terms of sub-G_1_ fraction, no difference was reported between ELF and sham-exposed cells, neither in 2D and 3D cultures (Fig. 3b). Concordantly, no activation of caspase-3 was triggered in response to 72 ELF-MF exposure in both 2D multi-wells and 3D matrix (Fig. 3d).

We also verified whether ELF-MF might stimulate invasiveness and neovascularization in neuroblastoma cells. The expression level of the hypoxia-inducible factor 1-alpha (*HIF-1α*), the vascular endothelial growth factor (*VEGF*), and the platelet-derived growth factor (both *PDGF-A* and *PDGF-B* variants) [53, 54] was screened in both 2D and 3D cultures. As reported in Fig. 3f, no change was induced in neuroblastoma cells by 72-h continuous exposure to 50-Hz MF.

### 50 Hz MF Does Not Alter the Expression of Neuroblastoma-Specific MicroRNAs

**Fig. 4:**
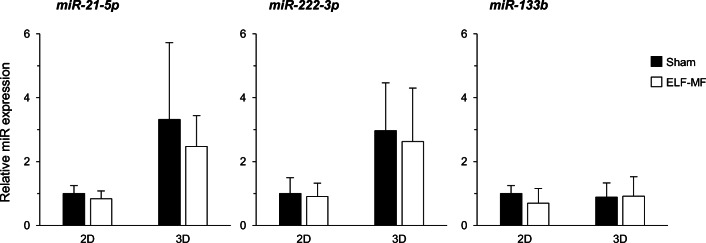
Exposure to 50-Hz MF does not alter the expression level of microRNAs in proliferating SH-SY5Y cells. Evaluation of the expression level of miR-21-5p, miR-222-3p, and miR-133b carried out in 2D- and 3D-cultured cells in response to ELF-MF (72 h of continuous exposure) by Exiqon-based real-time PCR (normalized to RNU1A1 small nucleolar RNA). Values are means ± SD (*N* = 3 independent experiments)

Epigenetic regulation, that also involves microRNAs (miRNAs) metabolism and function, affects several aspects of carcinogenesis, such as proliferation, invasion, and drug and radiation response of tumor cells, including neuroblastoma [55–57]. We here assessed the expression level of a set of neuroblastoma-specific miRs to verify whether they might be tuned by the magnetic field. As shown in Fig. 4, we evaluated miR-21-5p, miR-222-3p, and miR-133b by real-time PCR and demonstrated that no significant change occurred in their expression in SH-SY5Y cells in response to 50-Hz MF exposure if compared to sham, neither in the 2D nor in the 3D experimental conditions.

### 50-Hz MF Drives Glutathione Depletion and *SOD1* Deregulation Exclusively in 3D-Cultured SH-SY5Y Cells

**Fig. 5:**
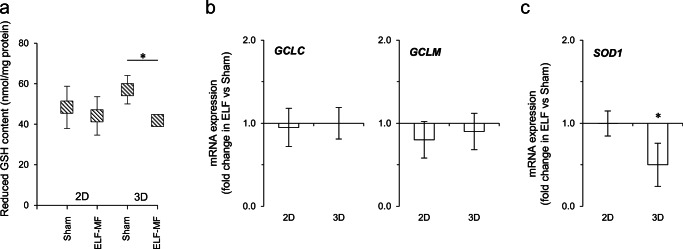
50-Hz MF triggers GSH content depletion and *SOD1* transcript deregulation exclusively in 3D-cultured SH-SY5Y cells. Cells were exposed for 72 h to 50-Hz (1 mT) MF and characterized in terms of **a** the intracellular reduced GSH content. Values are means ± SD (*N* = 3 independent experiments); *P < 0.05; **b**, **c** gene expression analysis of redox-related enzymes (normalized to 18s expression by real-time PCR). The values reported represent the mean fold change (± SD, *N* = 3 independent experiments) in mRNA expression of ELF- versus sham-exposed cells (chosen as a reference value). *P < 0.05 in the in ELF-MF vs sham-exposed cells

In conventional 2D cultures, we previously demonstrated that 50-Hz MF triggers thiol depletion and reactive oxygen species increase in both proliferating and differentiated SH-SY5Y cells [31, 33, 34]. We here extended the redox characterization by analyzing additional endpoints in 3D compared to 2D cultures. As reported in Fig. 5a, the intracellular reduced glutathione (GSH) content underwent a significant decrease in ELF- versus sham-exposed cells (72-h exposure), exclusively in Alvetex^®^ scaffold. This depletion was not accompanied by any change in the gene expression level of the rate-limiting enzyme controlling glutathione synthesis, namely the glutamate-cysteine ligase (*GCL*). We indeed assayed both GCL chains, i.e., the catalytic (GCLC) and the modifier (GCLM) subunits as they are often differentially regulated [58], and reported no significant modification in response to ELF-MF (Fig. 5b). We also monitored the superoxide dismutase 1 (*SOD1*) mRNA level and demonstrated that its expression underwent significant decrement following ELF exposure exclusively in the 3D matrix, whereas no change was observable in conventional plates (Fig. 5c).

### 50-Hz MF Stimulates the Dopaminergic Differentiation of SH-SY5Y if Grown in 3D Cultures

**Fig. 6:**
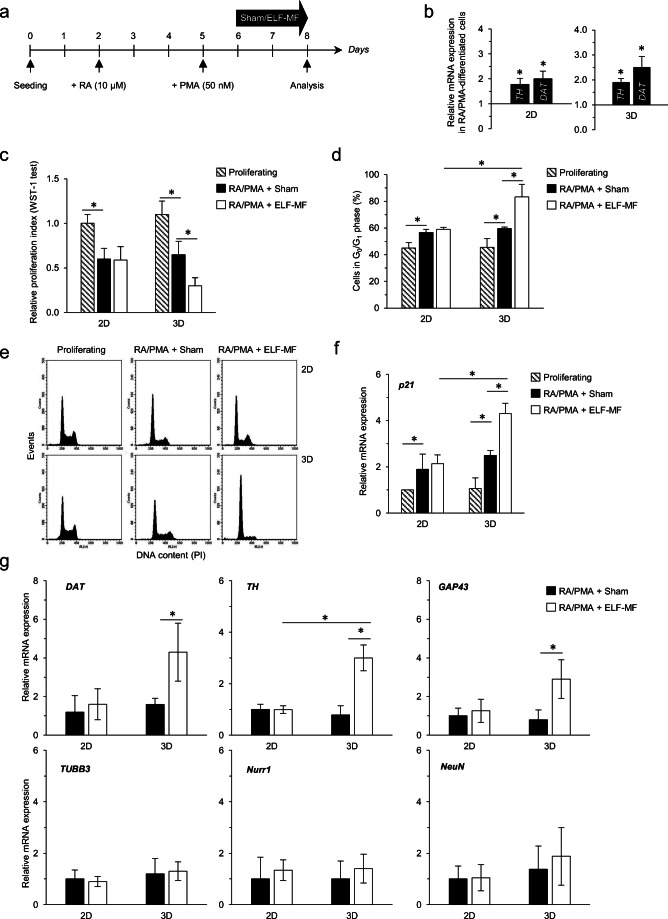
Exposure of 3D-grown SH-SY5Y neuroblastoma cells to ELF-MF stimulates the differentiation into a dopaminergic (DA) phenotype. **a** Graphical sketch of the experimental schedule. SH-SY5Y cells were seeded in either 2D multi-wells or 3D Alvetex^®^ scaffold and treated as follows (DA differentiation + MF exposure): 3 days of RA administration (without any MF/sham exposure), followed by 3 days of PMA treatment (the latter 2 days in the presence of either ELF-MF or sham exposure). **b** The expression level of *TH* and *DAT* was assessed in both 2D and 3D growing conditions at the end of the differentiation treatment (RA + PMA, without any MF exposure) compared to the basal proliferating phenotype. Values are means ± SD (*N* = 3 independent experiments); *P < 0.05, calculated in the RA/PMA-differentiated cells versus the proliferating ones (chosen as reference). Evaluation of the **c** relative proliferation index (by WST-1 assay), **d** percentage of cells populating the G_0_/G_1_ phase of the cell cycle with **e** corresponding FACS histograms chosen as representative of three independent experiments. **f** Analysis of the *p21* transcript expression level, carried out by real-time PCR (18s normalization). All analyses in panels **c–f** were performed at the end of the differentiation/exposure schedule. Values represent means ± SD (*N* = 3 independent experiments); *P < 0.05. **g** Gene expression assessment of *DAT*, *TH*, *GAP43*, *TUBB3*, *Nurr1*, and *NeuN* carried out by real-time PCR (18s normalization) in the RA/PMA-differentiated cells following exposure to either 50 Hz or sham field. Values are means ± SD (*N* = 3 independent experiments); *P < 0.05

Whether ELF-MF can stimulate or inhibit neuroblastoma differentiation is still controversial [59–61]. We thus assessed the response of differentiating SH-SY5Y cells to 50-Hz MF in 3D compared to 2D conditions. The differentiation/exposure schedule has been previously optimized by our group [31, 33] and summarized in Fig. 6a: neuroblastoma cells underwent differentiation toward a dopaminergic (DA) phenotype by a combination of retinoic acid (RA, for 3 days) plus phorbol 12-myristate 13-acetate (PMA, for 3 days) [44, 45], the latter administered in combination to either ELF-MF or sham exposure over the final 48-h period.

In both 2D and 3D culture conditions, RA/PMA treatment drove cell differentiation, as demonstrated by the stimulation of DA-specific biomarkers (dopamine transporter-*DAT* and tyrosine hydroxylase-*TH* genes) (Fig. 6b) and reduced proliferation rate (WST-1 test, Fig. 6c: comparison between proliferating and RA/PMA + sham). Consistently, cells accumulated in G_0_/G_1_ phase of the cell cycle (Fig. 6d–e) and stimulated the expression of *p21*^*CIP*^ gene (Fig. 6f) in response to the differentiating agents if compared to the proliferative condition.

Interestingly, once exposed to the 50-Hz MF, cells activated a different response according to culture conditions. In conventional 2D monolayers, ELF exposure did not alter the differentiative pathway, as proliferation index, G_0_/G_1_ cell percentage and *p21*^*CIP*^ accumulation overlapped sham-exposed cells (Fig. 6c–f). By contrast, the exposure to ELF-MF enhanced the differentiation pattern when cells were grown in 3D scaffold. Under this experimental condition, the proliferation index was significantly reduced if compared to sham (Fig. 6c) exclusively in the 3D culture condition. Accordingly, ELF-treated cells further accumulated in the G_0_/G_1_ phase of the cell cycle and a small but significant increase in *p21*^*CIP*^ expression level was detectable exclusively in the Alvetex^®^ scaffolds (Fig. 6d–f).

We finally evaluated the expression of both DA-specific and neuronal biomarkers in response to magnetic stimulation. As reported in Fig. 6 g, exposure to 50-Hz MF enhanced the expression of *DAT*, *TH*, and *GAP43* exclusively in the SH-SY5Y cultured in 3D matrix. No change between sham and ELF-MF was observed in cells grown in conventional plates. For both conditions, we did not report any statistical difference in *TUBB3*, *Nurr1*, and *NeuN* expression levels (Fig. 6g).

We also proved that the here-reported protocol, which combines differentiation and MF exposure (according to Fig. 6a), is the only schedule triggering a significant stimulation of the differentiation by ELF-MF. Two other combinations have been tested (Online Resource 4), carried out either by exposing cells together with RA agent (schedule i) or by pre-treating SH-SY5Y cells with 50 Hz for 48 h followed by RA/PMA differentiating agents (schedule ii, Online Resource 4a). Under both experimental conditions, ELF-MF was not able to affect the differentiation of SH-SY5Y cells grown in both 2D and 3D culture conditions, as proliferation index (WST-1 test, Online Resource 4b), G_0_/G_1_ phase percentage, and cell cycle distribution (Online Resource 4c,d) did not undergo any change in ELF- versus sham-exposed cells.

## Discussion

### Three-Dimensional (3D) Cultures Are Better Experimental Models for Studying the Neuronal Response to ELF-MFs than Conventional 2D-Cultures

We here demonstrate that the 3D culture of SH-SY5Y human neuroblastoma cells is a more reliable experimental model for studying cell response to ELF-MF if compared to 2D conventional monolayer, as it allows the identification of cellular and molecular events that might be underestimated or missing in 2D growing conditions.

We report that the antioxidant defense and the differentiating ability of neuroblastoma cells are specifically modulated by ELF-MF exclusively if cultured in 3D matrixes, thus supporting the concept that a 3D environment is an added value to model the key features of such response to non-ionizing radiations. Our data support what already suggested by literature, stating that 2D models do not fully recapitulate the *in vivo* physiology as they fail to reproduce the architecture and the possible connections characterizing a tissue. The *in vitro* 3D cultures can help overcoming these limitations and, compared to *in vivo* models, are promising tools for large-scale screening, are less expensive, and allow the growing and characterization of human cells.

Herein, we report that no significant changes occur during exponential growing phase in terms of shape, proliferation, cell cycle distribution, and expression of genes controlling cell cycle duplication and differentiation if cells are grown in 3D compared to 2D. We focused on such proliferation biomarkers, as residential exposure to ELF-MF has been mainly associated with increased risk for childhood leukemia and glioma; these radiations have been indeed classified as possibly carcinogenic to humans by the International Agency for Research on Cancer (IARC, 2002) [62]. We cannot exclude that the global gene expression profile of SH-SY5Y cells might be affected by growing condition in 3D Alvetex^®^ scaffold compared to 2D, as previously characterized—mainly in terms of neuronal morphology and neurite outgrowth—by other authors in different matrices like collagen I, Matrigel, and innovative eumelanin-coated polylactide microfibers [10, 63]. These authors performed gene expression profiling of SH-SY5Y cells after 24 h of 3D culture [10] and assessed the neuronal features after 7 (and up to 21) days in culture to suggest that 3D structure was able to decrease proliferation and promote differentiation [63]. We tested growing phenotype after 4 days in both 2D/3D cultures to characterize phenotype over a time window that might be consistent with the following ELF exposure setup and reported no significant changes.

3D cultures have been mainly developed in the field of the experimental oncology to screen cancer cell response to drugs and radiations [13–17, 64]. To the best of our knowledge, our data are the first experimental findings addressing the issue of neuroblastoma response to 50-Hz MF in a 3D model compared to conventional 2D monolayers.

Dosimetric computations carried out in 3D samples demonstrated that the level of induced E-field and current density displays a high homogeneity, hence guarantying a well-controlled and reproducible exposure condition.

Under this exposure modality, we demonstrate that long-term (72 h) continuous exposure to ELF-MF does not drive any change in proliferation and apoptosis activation in SH-SY5Y cells, independently of their 2D/3D growing conditions. Experimental data in 2D are consistent with what was previously reported by our group in neuroblastoma cells, as we already demonstrated that 50-Hz (1 mT) MF does not affect proliferation and death if applied for 72 h [31]. We here prove that, even if cultured in 3D conditions, SH-SY5Y cells do not undergo any significant modulation in cell cycle distribution and expression of proliferative biomarkers. Moreover, we included a set of invasiveness and pro-angiogenetic biomarkers (*HIF-1α*, *VEGF*, and *PDGF*), to verify whether ELF exposure might modulate neovascularization; malignant neuroblastoma is a highly vascularized solid tumor that requires access to blood vessels for growth, invasion, and metastasis [53, 54]. Previous findings addressed the issue of a possible ELF-MF-dependent effect on neovascularization and angiogenesis [65], but never in neuroblastoma cells. Under our experimental conditions, we did not report any significant change in all gene expression following 72-h ELF exposure. As a possible future perspective, the medium obtained from ELF-exposed SH-SY5Y might be administered to endothelial cells *in vitro* (or in a co-culture setup) to verify whether factors released from neuroblastoma cells might affect the proliferation of vascular cells.

The pro-oxidant ability of ELF-MF is a well-documented event in both *in vitro* and *in vivo* experimental models of neuronal and neuroblastoma cells [39–42], as also reported by our group [31, 33, 34]. We used such biological endpoint as a proof of concept for testing the reliability of 3D cultures. We extended the characterization to molecules that we did not previously test and demonstrated that the intracellular antioxidant defense, in terms of reduced GSH content and *SOD1* expression, was significantly impaired in ELF-exposed cells only if cultured in 3D scaffold. These findings open the question on whether 2D monolayers allow the complete characterization of the pathways tuned by the radiation, as there might be biological events that are missing or underestimated if cells are grown in 2D cultures. Redox impairment is a key event driven by ELF-MF in neuronal cells, and it has been identified as a possible candidate mechanism underlying the neurodegenerative potential of ELF-MF. Epidemiological data have indeed suggested a possible association between occupational and environmental exposure to ELF-MF with the increased incidence of neurodegenerative diseases, mainly ALS and AD [24, 25], where the oxidative stress–derived cellular damage is a master pathogenic event. The possibility to extend such redox characterization in 3D models might be an added value to deepen the mechanisms underlying the interaction between the electromagnetic field and the biological context.

Epigenetic plasticity is an essential regulatory level forging the cancer cell phenotype and response to therapy, including neuroblastoma [66]. Among the different patterns of epigenetic regulation, microRNA-dependent pathways have been reported to drive sensitivity to ionizing radiations in neuroblastoma [67, 68]. In terms of biological response to ELF-MFs, we recently demonstrated that miR-34b/c is involved in the response of SH-SY5Y cells to 50-Hz MF through a DNA methylation–dependent promoter regulation [33], but the whole epigenetic characterization of such response still has to be fully defined. We here extended the profiling of miR expression to a set of neuroblastoma-specific microRNAs (miR-21-5p, miR-222-3p, miR-133b) associated with neuroblastoma cancer progression and invasiveness [55–57], and demonstrated that they are not affected by 72 h of continuous ELF exposure, independently of cultures conditions. This finding might be consistent with the lack of morphology and proliferation changes here reported, as these microRNAs often target genes specifically devoted to cell cycle control and neuronal growth [69–71].

### ELF-MF stimulates the RA-PMA-driven differentiation of the SH-SY5Y neuroblastoma cells

Whether ELF-MF can stimulate or inhibit neuroblastoma differentiation is still controversial. In 2D conventional cultures, long-term exposure (192 h) to 50-Hz (1 mT) MF displayed a neuronal differentiating potential in SH-SY5Y [60]. In combination experiments (ELF plus RA), exposure to the MF (50 Hz, 1 mT for 72 h) showed an antagonistic effect against the RA-mediated differentiation on LAN-5 human neuroblastoma cells [59], whereas it drove a synergic effect of RA on BE(2)C human neuroblastoma cell line [61]. By means of a 3D scaffold model, we here demonstrate that 50-Hz MF enhances the differentiating potential of the RA + PMA combination in SH-SY5Y cells. The stimulation of the differentiation phenotype is exclusively reported in the 3D culture, further supporting the added value represented by the 3D scaffold compared to monolayers. Moreover, the synergistic effect is observed only when the MF is applied at the end of the differentiation protocols. No comparable effect has been proven when ELF is concomitantly administered with the RA or if given before treating cells with the differentiating agents. These experimental pieces of evidence open the way to future investigation for the characterization of the molecular mechanism(s) underlying such ELF-RA/PMA synergic effect specifically demonstrated in 3D cultures. The order of combination matters, thus suggesting that specific pathways are tuned by the MF in order to stimulate the differentiation in a 3D pattern. A biological hypothesis might involve the stimulation of plasma membrane receptors by ELF-MF. By applying a combined macroscopic and microscopic dosimetric evaluation, we recently demonstrated that increased current densities are induced at the plasma membrane/extra-cellular medium interface in SH-SY5Y cells in response to 50-Hz MF, thus identifying the plasma membrane as the main site of the ELF-neuroblastoma cell interaction [34]. We also proved that the membrane NADPH oxidase (Nox) is a direct target of the ELF-driven redox imbalance in SH-SY5Y cells [34], as Nox activity is stimulated by the exposure to ELF-MF. The involvement of Nox in the RA-induced differentiation has been reported in SH-SY5Y [72]; by pharmacologically inhibiting Nox activity, the authors demonstrated that RA acts through this enzyme to induce morphological changes typical of cell differentiation. ELF-MF might act at Nox level, thus mimicking a differentiation agent; besides, as neuronal differentiation is associated with fine regulation of redox-related pathways [73, 74], the well-characterized ELF-induced redox imbalance might contribute to the enhanced differentiation ability. Additionally, ELF-MF might tune other plasma membrane proteins, like receptors and ion channels, to help differentiation. Differentiation agents have been proven to stimulate plasma membrane receptors in SH-SY5Y cells, such as NRP1, PLXNA2, PLXND1, and Trk receptors [75], as well as ion channels. Differentiation of neuroblastoma cells has been associated with a remodeling of the store-operated Ca^2+^ entry, mediated by changes in the expression of calcium release–activated calcium pore proteins [76, 77]. Interestingly, calcium channels have been reported to be regulated by ELF-MF exposure in neuronal cells [78, 79], thus suggesting a further level of regulation of the differentiation pattern possibly elicited by ELF. Finally, the use of alternative neuronal experimental models, to be characterized in 3D culture conditions, might provide additional clues to further demonstration of this MF-dependent biological effect.

As overall, our findings characterize for the first time the response of SH-SY5Y neuroblastoma cells to 50-Hz, 1-mT MF in an *in vitro* 3D culture model and demonstrate that ELF-MF triggers oxidative stress and synergizes with RA and PMA to drive differentiation, without affecting miR-21-5p, miR-222-3p, and miR-133b expression. We also prove the limitations of conventional monolayer cultures for an exhaustive identification of pathways underlying the neuroblastoma response to ELF-MF and put the bases for promoting the use of 3D cultures in future experimental studies addressing the interaction between electromagnetic fields and biological systems.

## Data Availability

All data generated or analyzed during this study are included in this article and in the supplementary information

## Abbreviations

2D: Two-dimensional
3D: Three-dimensional
AD: Alzheimer’s’ disease
ALS: Amyotrophic lateral sclerosis
B-field: Induction magnetic field
E-field: Electric field
ELF-MF: Extremely low-frequency magnetic field
H&E: Hematoxylin and eosin
HIC: Immunohistochemistry
IF: Immunofluorescence
J: Current density
MF: Magnetic field
PMA: Phorbol 12-myristate 13-acetate
RA: Retinoic acid
RMS: Root mean square
WST-1: Water Soluble Tetrazolium Salt-1
